# Supplementary figures and images for: Spatial distribution and characteristics of women reporting cervical cancer screening in Malawi: An analysis of the 2020 to 2021 Malawi Population-based HIV Impact Assessment survey data
Source: PLoS One. 2024 Oct 10;19(10):e0309053. doi: 10.1371/journal.pone.0309053 (PMC11469604; doi:10.1371/journal.pone.0309053)

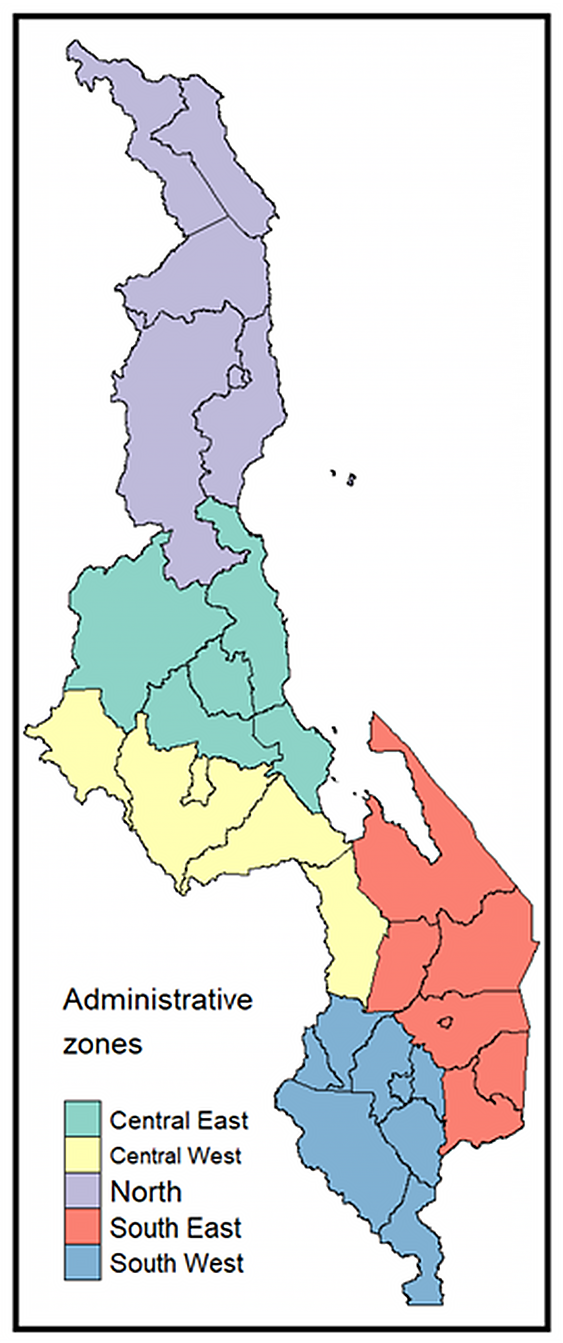

Supplement: S1 Fig — (TIF) [file pone.0309053.s001.tif]

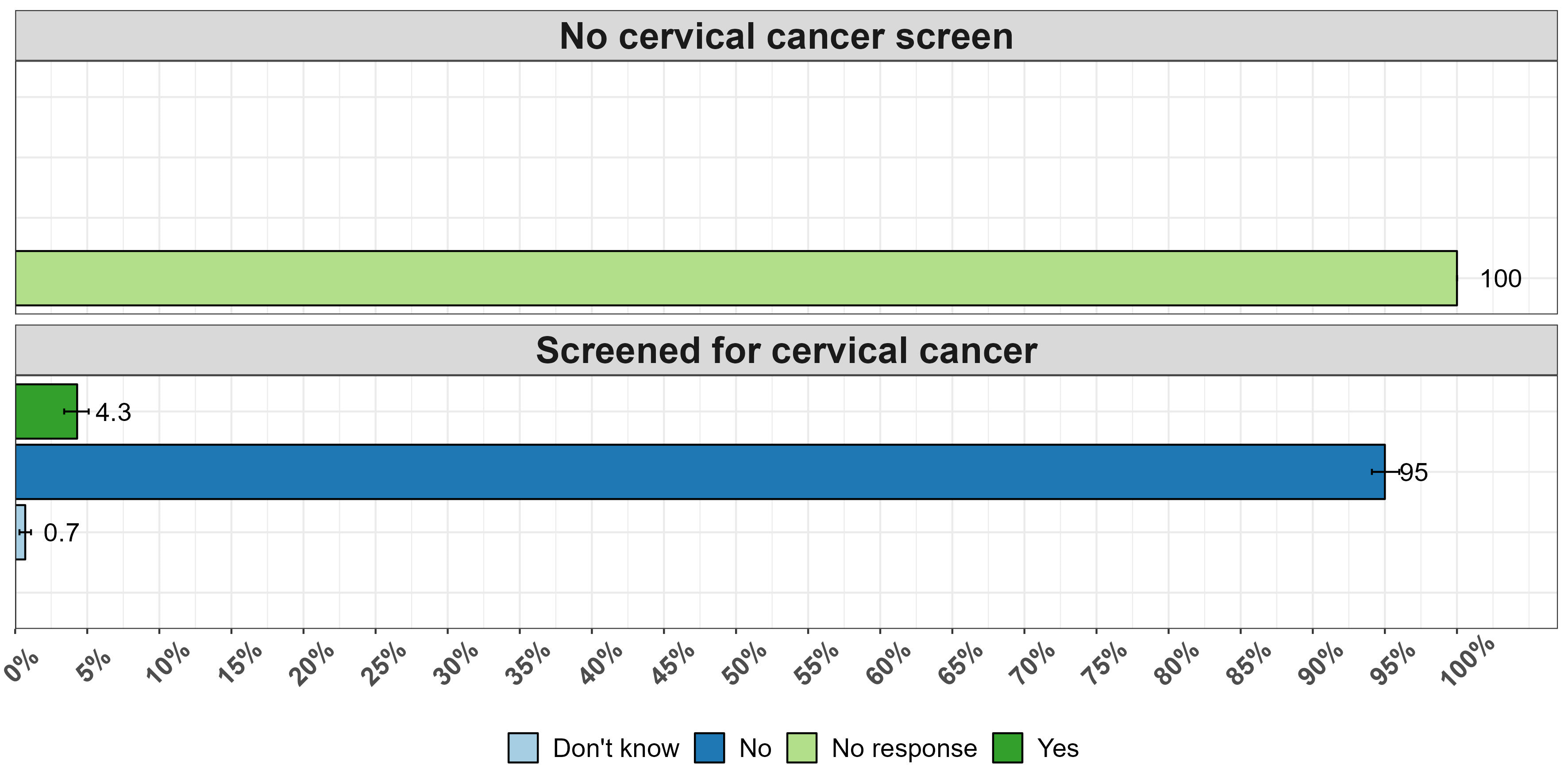

Supplement: S2 Fig — (TIFF) [file pone.0309053.s002.tiff]
