# Supplementary material for: Spatial distribution and characteristics of women reporting cervical cancer screening in Malawi: An analysis of the 2020 to 2021 Malawi Population-based HIV Impact Assessment survey data
Source: PLoS One. 2024 Oct 10;19(10):e0309053. doi: 10.1371/journal.pone.0309053 (PMC11469604; doi:10.1371/journal.pone.0309053)
Supplement: S1 Table — (DOCX) [file pone.0309053.s003.docx]

**S1 Table.** Variables included in the analysis

| **Variable** | **Definition** | **Type** | **Levels** | **Source** |
| --- | --- | --- | --- | --- |
| Age | Self-reported age in years | Numeric | Not applicable | MPHIA 2020 individual dataset |
| Age group based on CECAP targets | Age categorisation based on screening targets outlined in the National Cervical Cancer Control Strategy | Categorical | Less than 25 years; 25 to 49 years; 50 years and older | Derived from MPHIA 2020 |
| Age group based on WHO targets | Age categorisation based on screening targets outlined in the WHO Cervical Cancer Elimination Strategy | Categorical | Less than 35 years; 35 to 45 years; 46 years and older | Derived from MPHIA 2020 |
| HIV status | Final HIV status determination by MPHIA survey team based on self-report and test results | Categorical | HIV positive; HIV negative | MPHIA 2020 individual dataset |
| Ever been pregnant | Self-reported history of pregnancy | Categorical | Yes; No | MPHIA 2020 individual dataset |
| Geographical zone | As defined by household location | Categorical | North; Central East; Central West; Lilongwe City; Southeast; Southwest; Blantyre City | MPHIA 2020 individual dataset |
| Residence type | As defined by household location | Categorical | Rural; Urban | MPHIA 2020 individual dataset |
| Education | Self-reported highest level of education completed | Categorical | No education; Primary; Secondary; More than secondary | Derived from MPHIA 2020 individual dataset |
| Occupation | Self-reported occupation | Categorical | Unemployed; Mining; Transport; Construction; Uniformed personnel; Informal trade; Garment industries; Housekeeper; Student; Other | MPHIA 2020 individual dataset |
| Wealth quintile | As defined by the wealth index score | Categorical | Lowest; second; middle; fourth; highest | MPHIA 2020 individual dataset |
| Marital Status | Self-reported marital status | Categorical | Never married; Married or living together; Divorced or separated; Widowed | MPHIA 2020 individual dataset |
| Access to modern contraceptive methods | Defined by reported use of female sterilisation, the pill, intrauterine devices, injectable contraceptives, implants, or condoms | Categorical | Yes; No | Derived from MPHIA 2020 individual dataset |
