# Supplementary material for: Spatial distribution and characteristics of women reporting cervical cancer screening in Malawi: An analysis of the 2020 to 2021 Malawi Population-based HIV Impact Assessment survey data
Source: PLoS One. 2024 Oct 10;19(10):e0309053. doi: 10.1371/journal.pone.0309053 (PMC11469604; doi:10.1371/journal.pone.0309053)
Supplement: S2 Table — (DOCX) [file pone.0309053.s004.docx]

**S2 Table.** Variance inflation factors for independent variables considered for the multivariable model

| Variables | GVIF1 | GVIF2 |
| --- | --- | --- |
| HIV status | 1.349669 | 1.264220 |
| Age group | 2.872940 | 2.690473 |
| Geographical zone | 14.543971 | – |
| Residence type | 5.580117 | 1.854080 |
| Education | 3.355700 | 2.651392 |
| Occupation | 8.857303 | 4.889156 |
| Wealth quintile | 4.910465 | 3.400288 |
| Marital status | 3.643975 | 3.306795 |
| Ever pregnant | 2.195585 | 1.981429 |
| Access to modern contraceptive methods | 1.400506 | 1.341900 |
| *GVIF1 = generalized variance-inflation factors for model with Geographical zone;  GVIF2 = generalized variance-inflation factors for model without Geographical zone* | | |
