# Supplementary material for: Spatial distribution and characteristics of women reporting cervical cancer screening in Malawi: An analysis of the 2020 to 2021 Malawi Population-based HIV Impact Assessment survey data
Source: PLoS One. 2024 Oct 10;19(10):e0309053. doi: 10.1371/journal.pone.0309053 (PMC11469604; doi:10.1371/journal.pone.0309053)
Supplement: S3 Table — (DOCX) [file pone.0309053.s005.docx]

**S3 Table.** The proportion of women reporting vaccine uptake by age group

| Age group | Proportion vaccinated (95%CI) |
| --- | --- |
| 15-19 years | 16.9% (7.2, 26.7) |
| 20-24 years | 9.3% (5.4, 13.1) |
| 25-29 years | 4.7% (2.4, 7) |
| 30-34 years | 5.6% (3.3, 8) |
| 35-39 years | 3.1% (1, 5.2) |
| 40-44 years | 2.5% (0.9, 4.1) |
| 45-49 years | 1.7% (-0.3, 3.7) |
| 50-54 years | 0.6% (-0.5, 1.6) |
| 55-59 years | 1.5% (-0.6, 3.6) |
| 60-64 years | 0% (0, 0) |
| 65+ years | 1.3% (-1.1, 3.6) |
